# Foot traffic on turf primarily shaped the endophytic bacteriome of the soil-rhizosphere-root continuum

**DOI:** 10.3389/fmicb.2025.1488371

**Published:** 2025-04-09

**Authors:** Sayada Momotaz Akther, Jialin Hu, Grady Miller, Wei Shi

**Affiliations:** Department of Crop and Soil Sciences, North Carolina State University, Raleigh, NC, United States

**Keywords:** microbiome, endophytes, plant-microbial interaction, turfgrass, soil compaction

## Abstract

Foot traffic on turf can cause grass wear-stress and soil compaction, adversely impacting turf health. The root microbiome, consisting of diverse microbes, plays a crucial role in enhancing plant resilience to abiotic stressors. However, the effects of foot traffic on these microbes and the mechanisms they employ to help plant survival remain largely unknown. Here, we investigated how foot traffic affected microbial communities of the root endosphere, rhizosphere, and bulk soil in Bermudagrass (*Cynodon* spp.) and Zoysiagrass (*Zoysia* spp.) turfs. Foot traffic was simulated to mimic six professional football games per week using a modified Baldree traffic simulator. High-throughput amplicon sequencing targeting 16S rRNA for bacteria and ITS for fungi was employed to analyze microbial communities. Foot traffic slightly and significantly reduced soil moisture and inorganic nitrogen, likely due to soil compaction and associated impairment on microbial activity. Microbial alpha diversity varied across microhabitats, with no discernible effect of foot traffic. However, microbial community composition was impacted by foot traffic, being more pronounced on bacteria of the root endosphere and on fungi of the bulk soil. In light of the genetic potential predicted by PICRUSt2, foot traffic enriched a few pathways of the endophytic bacteriome, including nitrifier denitrification (PWY7084) and mannosylglycerate biosynthesis (PWY5656). This indicated that root endophytes could help turfgrass to tolerate foot traffic via controls on the concentration of nitric oxide, the signaling molecule for root growth, and mannosylglycerate, the compatible solute for protecting enzymes against osmotic stress. Foot traffic also enhanced degradation pathways of carbohydrates and 4-coumarate, the constituent of turfgrass cell walls (PWY-3801, PWY-2221, PWY-7046), indicating the faster turnover of root tissues. Along the root-rhizosphere-bulk soil continuum, the bacteriome varied substantially in composition and also exhibited contrasting genetic potentials from stress alleviation to nutrient supply in coping with grass growth. But foot traffic had little effect on the genetic potential of bacteriome in rhizosphere and bulk soil. Our findings indicated that the endophytic bacteriome was more sensitive to foot traffic than the bacteriome in the rhizosphere and bulk soil and could potentially help turf survival via influences on plant signal molecules and compatible solutes.

## 1 Introduction

Foot traffic on turfgrass, particularly in high-use areas like sports fields, inevitably leads to wear stress and soil compaction, significantly impacting turf health (Harivandi, 2002). On one hand, physical injury from pressure, shearing, abrasion, and tearing damages turfgrass, diminishing its aesthetic and functional qualities. On the other hand, foot traffic can lead to soil compaction and thus alter key soil physical properties, such as pore space, water infiltration, and aeration (Carrow and Wiecko, 1989; Carrow and Petrovic, 1992; Harivandi, 2002; Pagliai et al., 2003; Nawaz et al., 2013). Although compaction does not instantly kill turfgrass, it impedes growth and makes turfgrass more susceptible to various other stresses and harms over time (Carrow, 1995). Growing evidence indicates that plant-microbe interactions are salient to improve plant tolerance and adaptation to stress, yet the influence of foot traffic on microbial changes along the soil-rhizosphere-root endosphere continuum is largely unknown. This knowledge is needed, should sound anthropogenic interventions or management practices be informed to promote plant growth under stress conditions (Armada et al., 2018).

The soil-rhizosphere-root endosphere continuum features three consecutive yet functionally distinct microhabitats. The bulk soil is primarily involved in microbially-mediated nutrient transformations through the decomposition of organic matter (van der Heijden et al., 2008). By contrast, root exudates-rich rhizosphere, as well as root tissues, often host beneficial microbes to help defend against plant pathogens and enhance plant resistance to stresses (Compant et al., 2010). Consequently, not only do microbes at these microhabitats differ in community structure, but also their responses to environmental stimuli likely vary (Ding et al., 2019; Sun et al., 2021). Endophytic bacteria, sheltered from the high-stress conditions of the soil environment and in close contact with plant tissues, are believed to play significant roles in interacting with their host plants (Compant et al., 2005; Ryan et al., 2008; Gagné-Bourque et al., 2016). Our previous investigations on microbiomes in turf also revealed that grass root endophytes were more responsive to drought than microbes in bulk soil and the rhizosphere (Hu et al., 2023a,b).

Plants possess various mechanisms to cope with biotic and abiotic stresses, notably through symbiotic associations with microbes to govern phytohormonal production and signaling pathways (Liu et al., 2017; Jalmi and Sinha, 2022). A number of microbes, e.g., *Bacillus amyloliquefaciens*, *Pseudomonas fluorescens*, and *Bradyrhizobium japonicum* have been found to produce phytohormones such as indole-3-acetic acid (IAA), gibberellic acid (GA), and abscisic acid (ABA) to enhance root development (Boiero et al., 2007). Some microbes can also produce 1-aminocyclopropane-1-carboxylate (ACC) deaminase to degrade ACC, the precursor of ethylene, and therefore mitigate the inhibitory role of ethylene on root elongation (Gamalero and Glick, 2015), specifically when ethylene can be potentially and overly accumulated due to restriction of gas diffusion by soil compaction (Pandey et al., 2021). Microbes, e.g., *Azospirillum brasilense* can even enhance root colonization and lateral root formation through nitrite reductase activity, which produces nitric oxide (NO), crucial for auxin signaling (Creus et al., 2005; Lanteri et al., 2006). In addition, arbuscular mycorrhizal fungi (AMF) help host plants thrive under stress by enabling complex plant-fungus communication, which improves photosynthesis, gas exchange, and water and nutrient uptake (Birhane et al., 2012; Diagne et al., 2020). Morphologically, plants adapt to stress by widening root tips, secreting mucilage, and reshaping root architecture for better soil penetration (Materechera et al., 1992; Pandey et al., 2021; Huang et al., 2022). Nonetheless, plant adaptation strategies with the aid of the microbiome depend on environmental settings, plant genotype, and the type and intensity of stressors.

By physically wearing on leaves and vegetative bodies, foot traffic will reduce leaf area for photosynthesis but may prompt an increased proportion of photosynthates to be allocated to roots for nutrient uptake (Fohse et al., 1988; Poorter et al., 2012). Such a resource reallocation helps plants to select and interact with beneficial microbes and, in turn leverage microbial support for plant growth and stress resilience via mitigating nutrient deficiencies, improving water use efficiency, and enhancing photosynthetic traits, such as quantum yields of photosystem II (PSII), CO_2_ assimilation rate, stomatal conductance, and electron transport rate. Endophytes, like the fungus *Metarhizium brunneum* (Ascomycota) and bacteria *Providencia* sp. and *Proteus mirabilis* (both Proteobacteria), have been documented to be able to promote plant photosynthetic capability (Krell et al., 2018; Vishnupradeep et al., 2022).

In this study, we aimed to determine how microbiomes inhabiting bulk soil, rhizosphere, and root endosphere of the turf responded structurally and functionally to foot traffic. The investigation was conducted three seasons later after intensive foot traffic last fall. Zoysiagrass (*Zoysia japonica* Steud.; *Zoysia matrella* (L.) Merr.) and Bermudagrass (*Cynodon dactylon* (L.) x *C. transvaalensis* Burtt Davy) were chosen as model species because the two species fall in the category of “Excellent” in terms of their ability to tolerate wear stress from foot traffic (Carrow, 1995). This tolerance is often attributed to their rapid growth and high recuperative potential. However, the role of the root-associated microbiome is largely overlooked despite increasing evidence that plant-microbe interactions significantly contribute to the stress tolerance, growth, and overall health of plants. Our hypothesis was that root endophytes were particularly more sensitive to foot traffic than microbes in bulk soil and the rhizosphere due to a higher degree of proximity to foot traffic-induced changes in plant metabolism and, thus, phytochemicals. We also considered that microbial sensitivity to foot traffic manifested more on functional traits than taxonomy because the same species, defined as operational taxonomy unit with 99% similarity in 16S marker genes even, showed compartment (i.e., endosphere versus rhizosphere) biases in functional genes (Timm et al., 2015). By using 16S and ITS marker genes to identify bacteria and fungi and then using PICRUSt2 (Douglas et al., 2020) to predict microbial potential functions, we expected to provide insights into microbial strategies for mitigating foot traffic-induced stress in turfgrass systems.

## 2 Materials and methods

### 2.1 The study site and sample collection

The study site was located at the Lake Wheeler Turfgrass Field Laboratory of NC State University, where two grass species, Zoysiagrass (*Zoysia* spp.) and Bermudagrass (*Cynodon* spp.), had been subjected to foot traffic-induced soil compaction treatments (no foot traffic versus foot traffic) since August 2020. The experimental design employed a nested block structure, with the two grass species forming the primary blocks. The bermudagrass block included 12 cultivars or genotypes (Tifway, Tiftuf, Latitude36, OKC1873, OKC1876, OKC1666, OKC1406, OKC1682, MSB-1048, MSB-1075, MSB-1050, and MSB-1026), with each cultivar forming its own field plot. The zoysiagrass block also included 12 cultivars or genotypes (Meyer, Emerland, Zeon, FZ1410, DAIZ1808, DAIZ1806, DAIZ1802, DAIZ1714, DAIZ1808, DAIZ1311, DAIZ1408, and DAIZ1409) and each cultivar formed a field plot. These cultivar-based field plots were nested within the respective primary block and served as the experimental units for the two levels of foot traffic (i.e., foot traffic and no foot traffic). Foot traffic simulations were performed three times per week from September to November by using a modified Baldree traffic simulator (Kowalewski et al., 2013) to mimick the impact of six professional football games weekly. The study site was professionally managed for NPK fertilization and the use of herbicides, including oxadiazon, thiencarbazone, idosulfuron, and dicamba. Nitrogen was split-applied in spring at ~120 kg ha^−1^ yr.^−1^, and the preventative use of herbicides was often from January to June with oxadiazon, for example, being applied in late January and early February at ~3 kg ha^−1^ yr.^−1^.

To examine the legacy effects of annually periodic foot traffic on soil and phyto-microbiomes, an intact soil-grass core (4.5 cm dia. × 10 cm depth) was collected from each field plot right before foot traffic simulation being made in 2022, leading to a total of 48 sample cores. Low-temperature preservation method was used during sample transportation from the study site to the laboratory by an icepack-filled cooler and during sample storage before the separation of roots from rhizosphere and bulk soils by a 4°C refrigerator.

### 2.2 Root, rhizosphere, and bulk soil sample preparations

We followed a modified bleach-washing protocol (Xia et al., 2021) to prepare turfgrass root, rhizosphere, and bulk soil samples. Briefly, soil particles loosely attached to roots were collected as bulk soil samples. Rhizosphere soil samples were collected by shaking grass roots in a phosphate buffer (6.33 g/L NaH_2_PO_4_, 8.5 g/L Na_2_HPO_4_ anhydrous, and 200 mL/L Silwet L-77, pH 6.5), followed by centrifugation of soil slurries at 3,000 × g. The clean grass root samples were then obtained by sequential washing with a solution of 50% bleach containing 0.01% Tween 20, 70% ethanol, and sterilized water. At least two additional washes with sterilized water were made to help remove solvents. All apparatus involved were sterilized using 70% ethanol to prevent cross-contamination between samples, and all chemical solutions used for sample preparation were autoclaved. Grassroot, rhizosphere, and bulk soil samples were stored in a − 20°C freezer prior to DNA extraction. Additionally, aliquots of the bulk soil samples were kept at 4°C for the analysis of soil physicochemical properties.

### 2.3 Soil physicochemical properties

Soil gravimetric moisture content was estimated by differences in weight before and after oven drying at 105°C for 48 h. Soil pH was measured using a pH electrode (Fisher Scientific, Pittsburg, PA, United States) in a 1:2.5 soil (g)-to-water (mL) suspension. Soil NH_4_^+^-N and NO_3_^−^-N were quantified colorimetrically using the Berthelot reaction-based and Vanadium (III) chloride-based spectrophotometric methods, respectively (Doane and Horwáth, 2003) by a microplate reader (Biotic Instruments Inc., Winooski, VT, USA) after soil extraction with 2 M KCl at a 1:5 soil (g)-to-KCL (mL) ratio and the filtrate collected through a Whatman filter paper #42. Soil bulk density was also roughly estimated from the total weight and volume of intact soil cores by subtracting the weight and volume of rocks and plant roots.

### 2.4 DNA extraction, library preparation, and amplicon sequencing

The metagenomic DNA of bulk soil (~ 250 mg), rhizosphere soil (100–400 mg), and grass roots (100–200 mg) was extracted using DNeasy PowerSoil Pro Kits (Qiagen, Venlo, Netherlands) or FastDNA Spin Kit for soil (MP Bio, Solon, OH, United States), following the manufactures’ protocols. The concentration and the quality of DNA were then quantified and assessed using a NanoDrop Spectrophotometer (Thermo Scientific, Wilmington, DE, United States). Illumina-compatible adapter-added primer pairs, targeting the bacterial V3-V4 region (341F: 5’-CCTACGGGNGGCWGCAG-3′ and 805R: 5’-GACTACHVGGGTATCTAATCC3’) and fungal ITS1-ITS2 region (F_KYO2: 5′- TAGAGGAAGTAAAAGTCGTAA-3′ and R_KYO2: 5’-TTYRCTRCGTTCTTCATC-3′) were used to amplify DNA. PCR reactions were conducted in a 25-μL mixture comprising 12.5 μL of KAPA HiFi HotStart ReadyMix (KAPA Biosystems, Wilmington, MA, United States), 2.5 μL of template DNA, and 5 μL of forward and reverse primer (5 μM). For bacterial DNA amplification, the thermal cycling protocol consisted of an initial denaturation step at 95°C for 3 min, followed by 30 cycles of denaturation at 95°C for 30 s, annealing at 55°C for 30 s, and extension at 72°C for 30 s, and then a final elongation step at 72°C for 5 min. This protocol was also used for fungal DNA amplification yet thermal cycling conditions were modified as denaturation at 98°C for 30 s and annealing at 51°C for 15 s. Following purification with AMPure XP beads (Beckman Coulter Genomics, Danvers, MA, United States), DNA amplicons were barcoded using the Nextera XT Index Kit (Illumina, San Diego, CA, United States). This process was made in a 50 μL reaction mixture comprising 25 μL of KAPA HiFi HotStart ReadyMix, 5 μL of each forward and reverse index primer, 5 μL of purified PCR product, and 10 μL of PCR grade nuclease-free water. After undergoing a second round of purification with AMPure XP beads, DNA amplicons, now containing unique index sequences, were quantified using a NanoDrop spectrophotometer, diluted to a concentration of 40 nM, pooled together, and thoroughly mixed for sequencing on the Illumina MiSeq platform (300 × 2 PE, Illumina, San Diego, CA, United States) by Genomes Sciences Laboratory, NCSU. The sequences of 287 samples (i.e., the sample number of 2 grass species × 12 cultivars × 2 foot traffic treatments × 3 microhabitats × 2 kingdoms excluding one poor quality sample for bacteria) were deposited into the NCBI BioProject database with the accession number PRJNA1137239.

### 2.5 Bioinformatics and statistical analysis

Following demultiplexing, sequencing reads were cleaned by removing primers and the adapter using cutadapt (v1.18) (Martin, 2011). Then, clean reads were processed for dereplication, error model training, pair-end merging, and removal of chimeras (~ 10% on average) in R (3.6.1) using the DADA2 (v1.12.1) pipeline (Callahan et al., 2016; R Core Team, 2018) to generate a table of amplicon sequence variants (ASVs). After being imported into QIIME2 (v2023.2) and singleton removed, ASVs were classified as bacterial and fungal taxa against Greengenes (13_8) and UNITE (v9) databases, respectively. It is worth mentioning that prior to bacterial classification, sequences belonging to chloroplasts and mitochondria were removed from ASVs tables and representative sequences using the script --p-exclude chloroplast, mitochondria. Sequencing depth was rarified to 6,990 for 16S rRNA and 12,500 for the ITS region, and then metrics, including observed ASVs, Shannon index, Pielou’s evenness, and Chao1, were used to evaluate microbial alpha diversity. Bray–Curtis dissimilarity matrix was constructed for assessing microbial beta diversity; differences in microbial community composition were visualized by principal coordinate analysis (PCoA). Further, PICRUSt2 [Phylogenetic Investigation of Communities by Reconstruction of Unobserved States (Douglas et al., 2020)] was performed to estimate putative functional genes of bacterial metabolisms and processes using genes and copy numbers for KEGG ortholog identifiers and MetaCyc pathways.

Inferential statistics were performed to check if data met the assumptions of ANOVA, including normality using the Shapiro–Wilk test, the homogeneity of variance using Levene’s test, and residual independence by plotting residuals against independent variables. For parametric data (i.e., soil inorganic N, soil moisture, and soil pH), a linear mixed effects model (response ~ traffic + (1|species/cultivars)) with cultivars/genotypes nested within species was performed to test the significance of traffic treatments (traffic and control). For non-parametric data, including microbial diversity metrics and the relative abundance of microbial taxa, data was first evaluated for the type of distribution; then a generalized linear mixed effects model (response ~ traffic + microhabitats + traffic × microhabitats + (1|species/cultivars)) with beta distribution was performed to check statistical significance. Grass cultivars/genotypes were the random effect nested within species; traffic treatments and microhabitats were the fixed effects. If interactions exist between microhabitats and traffic treatments, multiple pairwise comparisons with Bonferroni adjusted *p*-values were used to compare no traffic and traffic differences across microhabitats. PERMANOVA (non-parametric permutational multivariate analysis) with adonis2 function and 999 permutations was performed to determine if treatments and microhabitats could significantly interpret variations in the microbial community. We also conducted a distance-based redundancy analysis (db-RDA) to compare the contribution and significance of soil properties and the traffic treatment to the divergence in the microbial community among samples of individual microhabitats. To assess differences in the relative abundance of KEGG genes and MetaCyc pathways between microhabitats and between traffic treatments, differential abundance method was employed with statistical significance being considered when the |log_2_-fold change| was greater than one and the adjusted *p*-value, false discovery rate (FDR) was less than 0.05. All statistical analyses were conducted using packages in R (v4.2.3), including fitdistrplus (v1.2.2), lme4 (1.1.36), glmmTMB (v1.1.10), Multcomp (v1.4.26), emmeans (v.1.10.6), vegan (v2.6–4), and edgeR (v3.40.2). A significance level of *P* or adjusted *p* < 0.05 was used unless stated otherwise.

## 3 Results

### 3.1 Effects of foot traffic on the microbial diversity of three microhabitats

**Figure 1:**
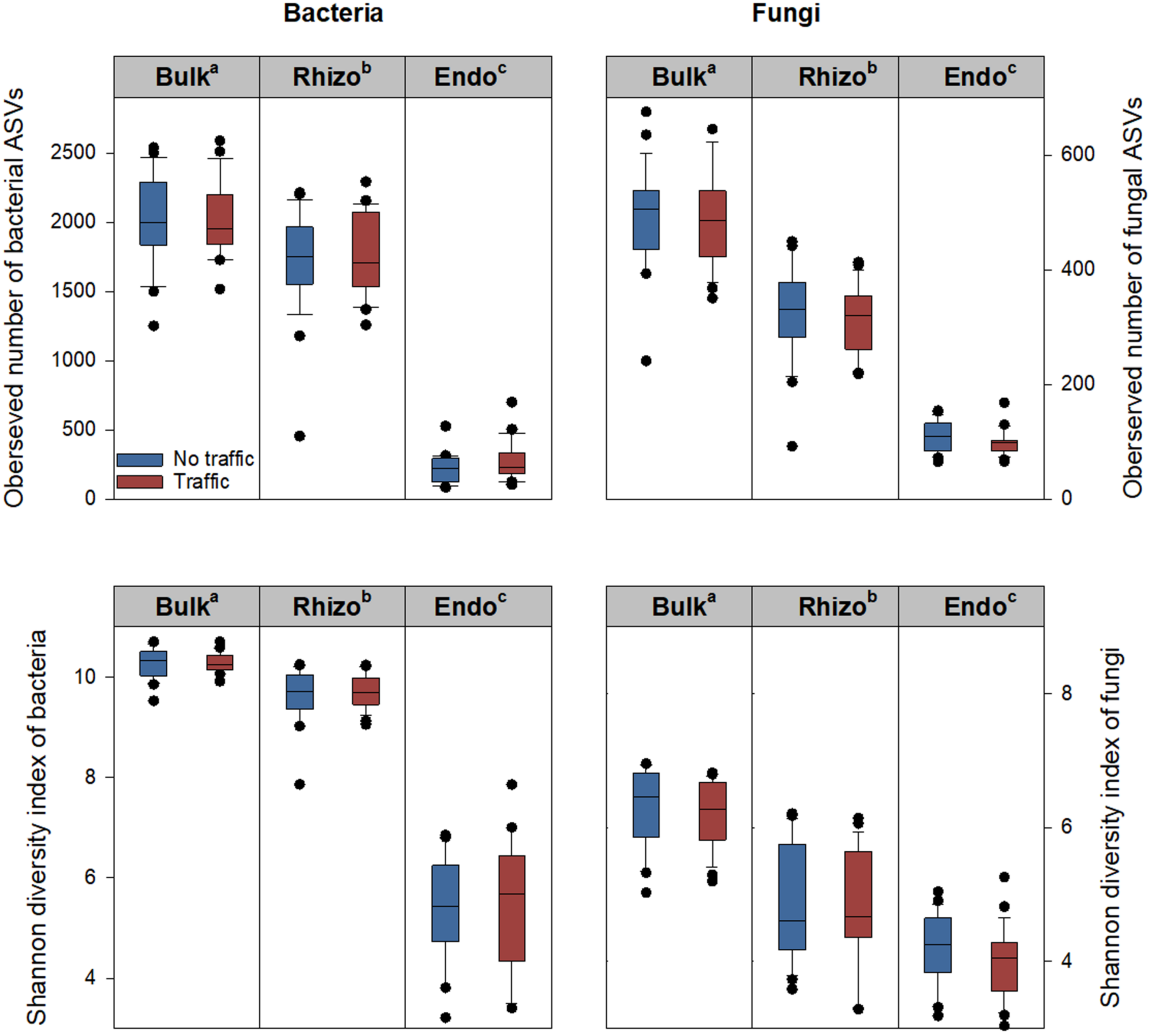
Bacterial and fungal alpha diversity metrics, including the observed number of ASVs (amplicon sequence variants) and Shannon index in three microhabitats (bulk soil, rhizosphere, and root endosphere) subjected to simulated foot traffic at three time per week from September to November, mimicking weekly six professional football games and no foot traffic treatment. The top, middle, and bottom lines of each box plot represent the upper 25% quartile, median, and lower 25% quartile of 24 replicates (i.e., 2 grass species × 12 cultivars). The sequencing depth was 6,900 and 12,500 for bacterial and fungal communities, respectively. Different letters labeling bulk soil (Bulk), rhizosphere (Rhizo), and root endosphere (Endo) indicate significant differences at *p* < 0.05 among three microhabitats.

The alpha diversity of bacterial and fungal communities was evaluated using five metrics, including the observed number of Amplicon Sequence Variants (ASVs), Pielou’s evenness, Shannon diversity index, Chao1, and Simpson index. Generally, the five metrics showed similar variation patterns among three microhabitats and between two traffic treatments. Therefore, only the observed number of ASVs and Shannon diversity index are reported (Figure 1). Significant differences were observed among microhabitats, being greatest in the bulk soil, followed by the rhizosphere, and least in the root endosphere. Compared to the bulk soil, the observed number of ASVs of the bacterial community was reduced by ~14% in the rhizosphere and ~ 88% in the root endosphere; respective reductions in the fungal community were ~ 39% and ~ 79%. The Shannon diversity index, which accounts for both the observed number and relative abundance of ASVs, also declined along the bulk soil-rhizosphere-root endosphere continuum. However, there were no differences in either the observed number of ASVs or the Shannon diversity index between traffic treatments (Figure 1).

**Figure 2:**
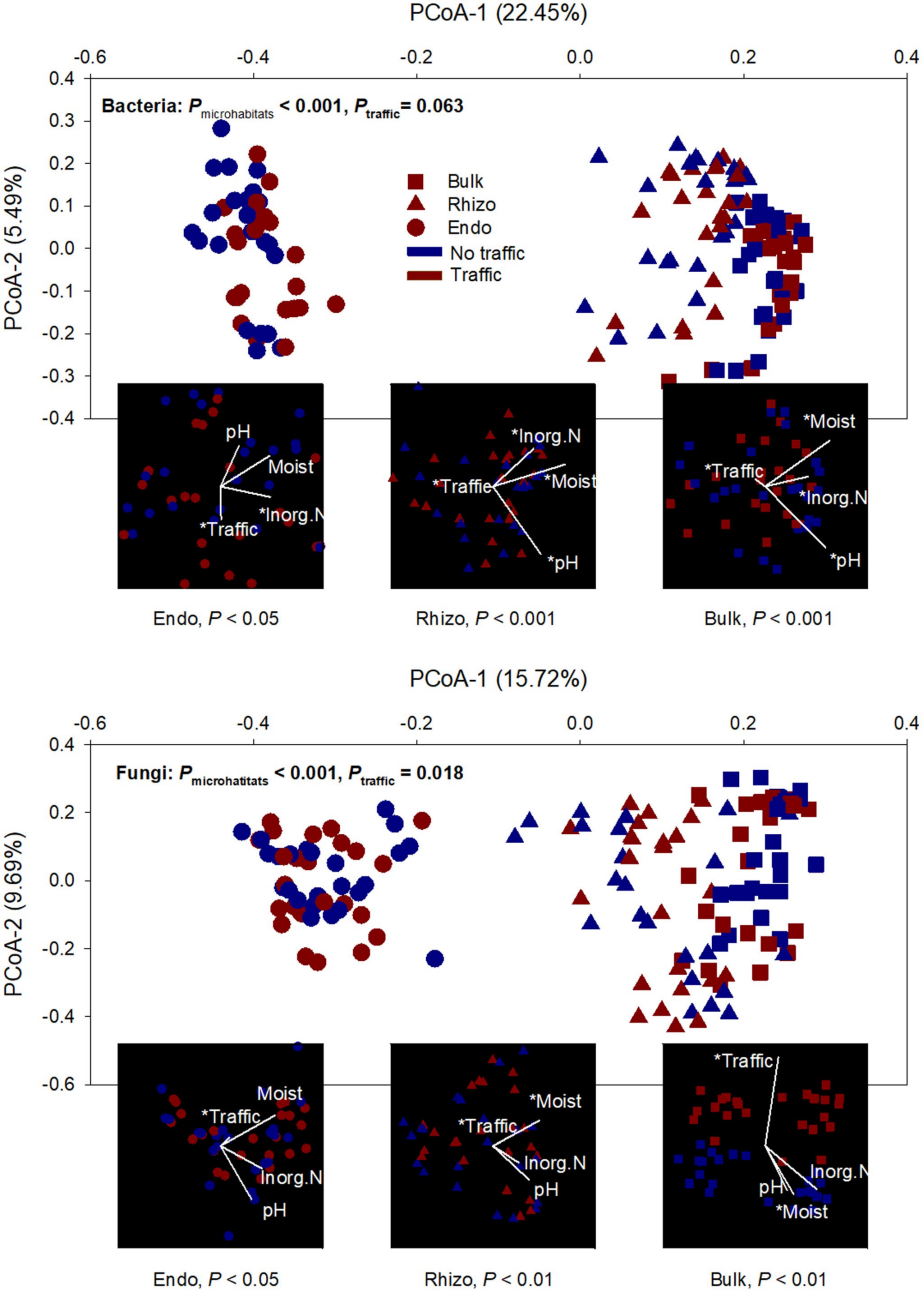
Ordination analyses of bacterial and fungal communities from three microhabitats (bulk soil, rhizosphere, and root endosphere) under simulated foot traffic at three time per week from September to November, mimicking weekly six professional football games and no foot traffic treatment. The PCoA (principal coordinate analysis) describes dissimilarities in the microbial community among microhabitats (Bulk: bulk soil; Rhizo: rhizosphere; Endo: root endosphere) and between foot traffic treatments. The combination of each level of microhabitat and foot traffic is represented by 24 replicates (i.e., 2 grass species × 12 cultivars). Variations explained by PCoA axes are given in parentheses. The embedded db-RDA (distance-based redundancy analysis) for each microhabitat describes the magnitude and direction of relationships between dissimilarities in the microbial community and soil properties and foot traffic treatments. The symbol * represents the significant relationship at *p* < 0.05.

Both bacterial and fungal beta diversity also varied considerably with microhabitats (Figure 2; Supplementary Table S1). Principal coordinates analysis (PCoA) revealed that the microbial community of the root endosphere formed a distinct cluster and was well-separated from those of the bulk soil and rhizosphere. However, foot traffic treatments did not significantly affect the fungal community (*p* = 0.069) or the bacterial community (*p* = 0.105, Figure 2; Supplementary Table S1). To better evaluate the contribution of foot traffic versus soil properties to variations in the microbial community, the distance-based redundancy analysis (db-RDA) was performed on the dataset of individual microhabitats. Across a total of 48 bulk soil samples, soil pH, inorganic N, and moisture varied moderately, i.e., < 31% CV (coefficient of variation, Table 1). We failed to reliably measure soil bulk density from both 0–5 cm and 5–10 cm depths, due to unavoidable and large experimental errors in preparing and quantifying root biomass. Nonetheless, all soil pH, inorganic N, and moisture were statistically greater (*p* < 0.01) in no-foot traffic than in the foot traffic treatment. For the bacterial community, db-RDA analysis showed that foot traffic significantly changed the beta diversity in all three microhabitats, yet the most pronounced effect appeared to be in the root endosphere (Figure 2). Changes in the beta diversity across the bulk soil-rhizosphere-root endosphere continuum were also associated with soil properties. Inorganic N was more important to the root endosphere, whereas soil pH dominated the rhizosphere and bulk soil. In contrast, for the fungal community, the effect of foot traffic was most pronounced in the bulk soil; soil moisture, rather than pH or inorganic N, was a significant soil property associated with changes in the beta diversity in the rhizosphere and bulk soil. None of the soil properties were significantly associated with changes in the beta diversity in the root endosphere.

**Table 1:** The statistical summary and residual effects of foot traffic on selected soil properties different letters indicate significant differences at *p* < 0.01.

	pH	Inorganic N (μg N g^−1^ soil)	Moisture (%)
Minimum	5.33	8.50	6.1
Maximum	6.70	34.36	30.7
Mean	6.05	23.63	15.0
SE	0.04	0.88	0.6
CV (%)	4.63	25.41	30.4
Treatments			
No-traffic	6.14a	26.16a	16.1a
Traffic	5.98b	21.31b	13.0b

### 3.2 Variations in microbial taxa among microhabitats and between traffic treatments

**Figure 3:**
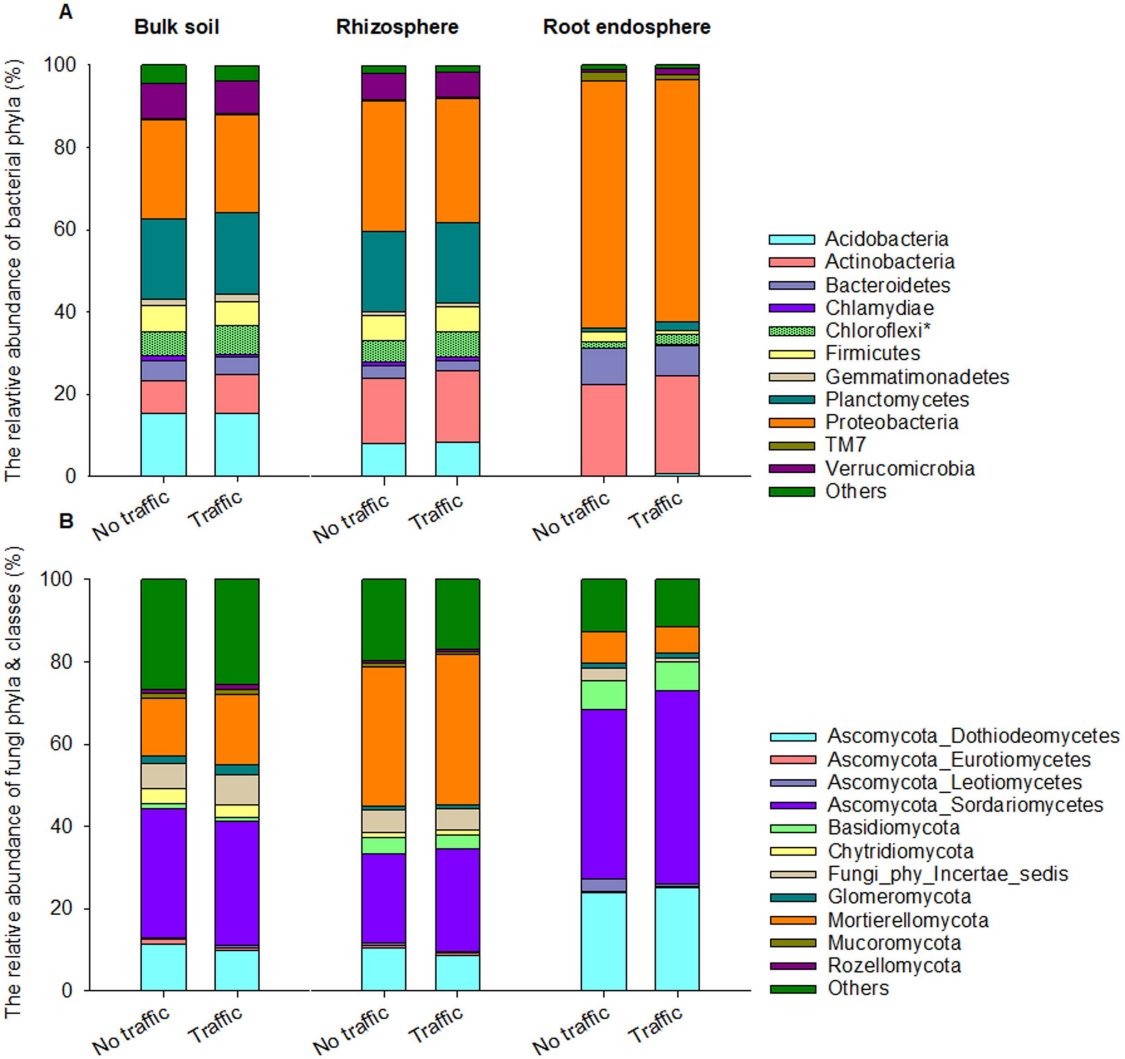
Abundant bacterial **(A)** and fungal **(B)** phyla across the bulk soil-rhizosphere-root endosphere continuum. Ascomycota, the fungal phylum is represented by four major classes, Dothideomycetes, Eurotiomycetes, Leotiomycetes, and Sordariomycetes. Bacterial and fungal taxa marked by the symbol * and bars with a texture denote significant differences in the relative abundance at *p* < 0.05 between simulated foot traffic at three time per week from September to November, mimicking weekly six professional football games and no foot traffic treatment. Each stack bar represents the mean value of 24 replicates (2 grass species × 12 cultivars).

Eight out of 45 detected bacterial phyla were dominant, having >1% relative abundance on average across three microhabitats. Proteobacteria, Actinobacteria and Bacteroidetes together accounted for ~90% of the total bacterial abundance in the root endosphere and were more abundant than those in the rhizosphere and bulk soil (Figure 3A). In contrast, Acidobacteria, Chloroflexi, Firmicutes, Plantomycetes, and Verrucomicrobia were more abundant in the rhizosphere and bulk soil than in the root endosphere. At the phylum level, we found that the relative abundance of Chloroflexi was slightly yet significantly greater under foot traffic than no-foot traffic across all microhabitats.

Regardless of microhabitats, the fungal community was dominated by Ascomycota, Basidiomycota, and Mortierellomycota, together accounting for ~74% of total fungal abundance on average (Figure 3B). Dothideomycetes and Sordariomycetes are the dominant classes of Ascomycota and were more abundant in the root endosphere than in the rhizosphere and bulk soil. Basidiomycota also exhibited a similar trend, with the relative abundance increasing from bulk soil to the root endosphere. On the contrary, Mortierellomycota was most abundant in the rhizosphere, followed by the bulk soil, and least in the root endosphere. Unlike bacteria, no fungal phyla responded significantly to traffic treatments.

**Figure 4:**
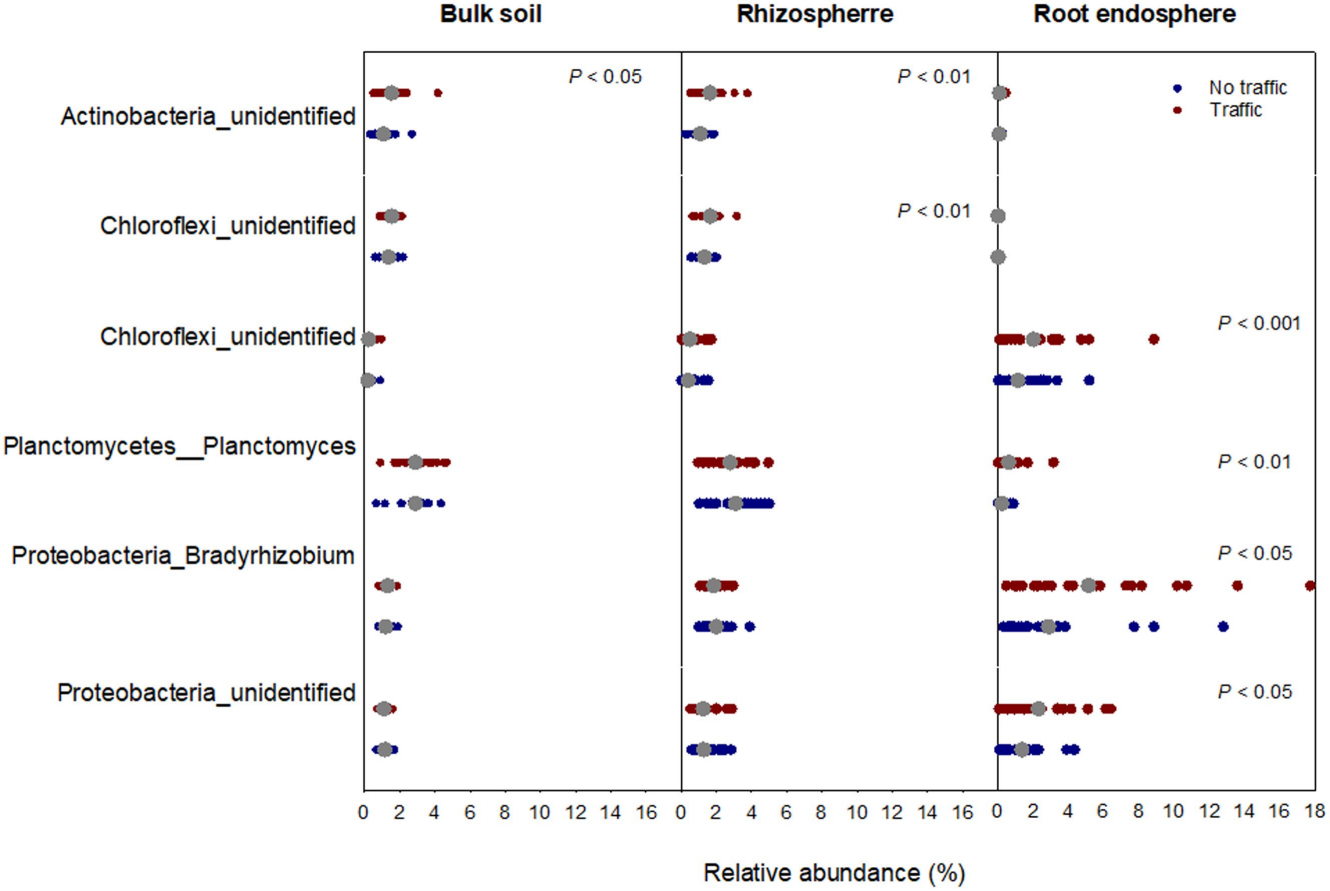
Foot traffic-sensitive bacterial genera in bulk soil (Bulk), rhizosphere (Rhizo), and root endosphere (Endo). Scatter plots represent the relative abundances of sensitive genera across all individual samples and their mean values (gray dots). Each scatter distribution represents 24 data points (i.e., 2 grass species × 12 cultivars). The Y-axis is labeled by phyla and genera together.

Down to the genus level, the effect of foot traffic on the bacterial community was more apparent for the root endosphere (Figure 4). A total of four genera belonging to phyla Chloroflexi, Plantomycetes, and Proteobacteria were detected to differ in the relative abundance between foot traffic and no-foot traffic in the root endosphere. This number declined to two in the rhizosphere and one in the bulk soil.

### 3.3 Foot traffic-responsive functional genes and pathways of the bacterial community in the bulk soil, rhizosphere, and root endosphere

**Figure 5:**
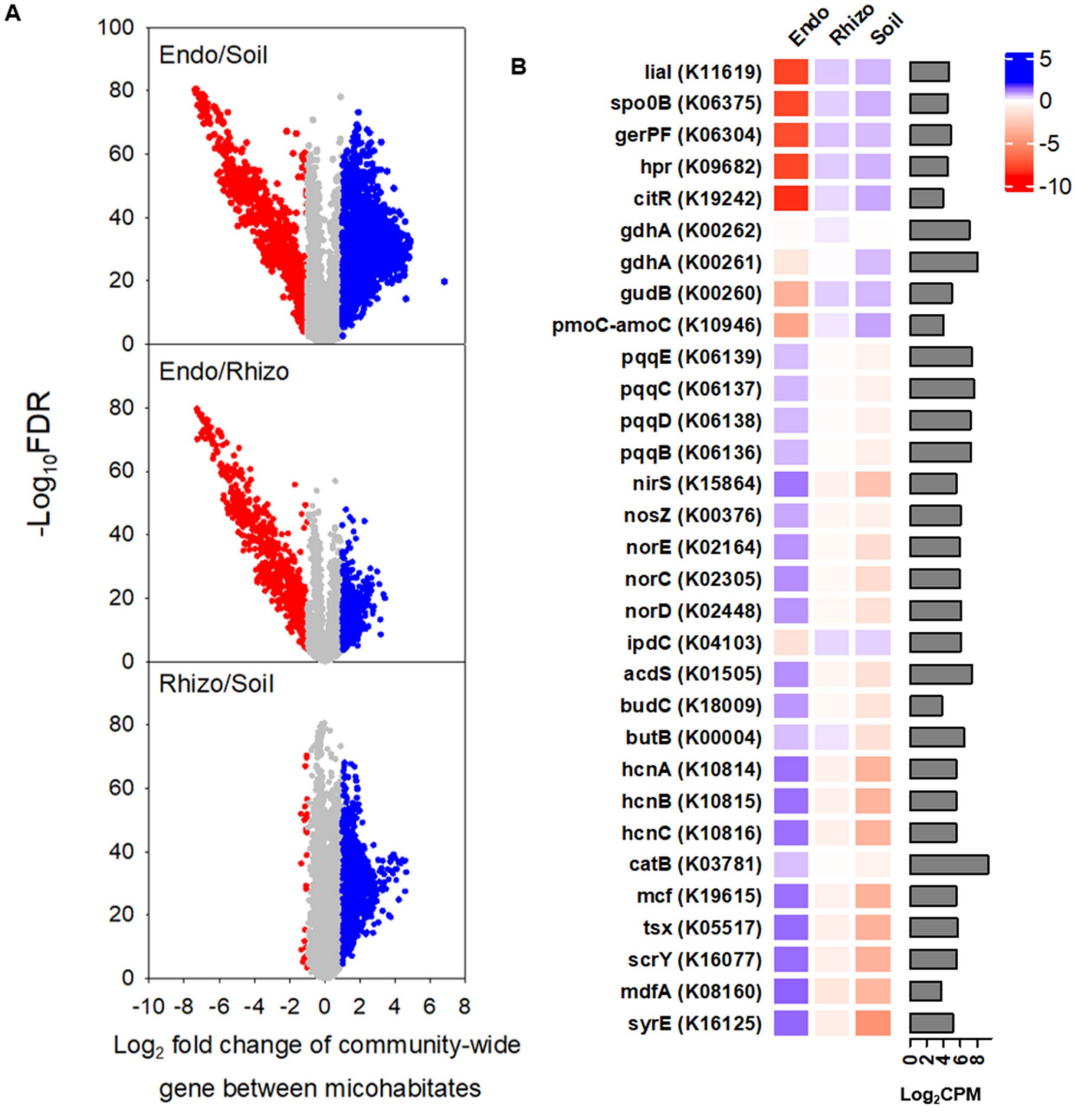
PICRUSt-predicted KO (KEGG orthology) genes in bulk soil (Bulk), rhizosphere (Rhizo), and root endosphere (Endo). **(A)** Volcano plots depict the significance and magnitude of changes in the relative abundance of KO genes between microhabitats. Each volcano plot represents 48 replicates (i.e., 2 grass species × 12 cultivars × 2 foot traffic levels). Gray represents no differences, whereas red and blue denote under-representation and over-representation, respectively, when comparing a microhabitat in nominator with that in denominator. Here, significant differences in the relative abundance of gene are defined as |Log_2_ fold change| > 1.00 and adjusted *P* (i.e., false discover rate) < 0.05. **(B)** The heatmap details significantly different genes in CPM (counts per million) between root endosphere and bulk soil (or rhizosphere) selected from the volcano plots, including the top five most under- and over-represented KO genes as well as genes contributing to plant stress adaptation. The bar plot is for the relative abundance of genes, averaged across all three microhabitats (i.e., 144 data points = 2 grass species × 12 cultivars × 2 foot traffic levels × 3 microhabitats), expressed as log_2_CPM.

Of a total of 4,441 putative functional genes with the relative abundance of >10 counts per million (CPM) on average across three microhabitats, ~ 50% or 35% in the root endosphere was found to differ significantly in the relative abundance from that in the bulk soil or rhizosphere, respectively (Figure 5A). However, most functional genes (i.e., ~ 82%) were similar in the relative abundance between the rhizosphere and bulk soil. The top five genes of lower abundance in the root endosphere versus bulk soil/rhizosphere were involved in sporulation and germination (spo0B, gerPF, hpr), bacterial envelope protection under stress (lial), and transcriptional regulation (citR) (Figure 5B). By contrast, the top five genes of the higher abundance were for toxin production (syrE, mcf) and efflux and influx transportations (mdfA, scrY, tsx). To better understand microhabitat-specific microbial functions, we further compared a breath of functional genes from nutrient transformations to plant stress adaptation. Genes encoding enzymes for nitrogen mineralization and nitrification (gdhA, gudB, pmoC-amoC) were more abundant in the rhizosphere and bulk soil than in the root endosphere. On the contrary, the root endosphere possessed more abundant genes contributing to phytohormone regulation (*acds*: 1-aminocyclopropane-1-carboxylate deaminase enabling degradation of ethylene precursor), immunity buildup (*budCB*: acetoin and 2,3-butanediol for induced systemic resistance), antimicrobial compounds (*hcnABC*: hydrogen cyanide for synthesis of antimicrobial compounds), signal molecules (*nirS, norECD, nosZ*: cytochrome cd1 nitrite reductase, NO reductase, N_2_O reductase, respectively, for the formation and consumption of NO, the root-branching signal), phosphorous acquisition (*pcqqBCDE*: pyrroloquinoline quinone-encoding genes for mineral phosphate solubilization), and oxidative stress regulations (*catB*: encoding catalase to detoxify H_2_O_2_). The relative abundance of *ipdC,* encoding for indole-3-pyruvate decarboxylase for the synthesis of the auxinic phytohormone indole acetic acid, was exceptional, being lower in the root endosphere than in the rhizosphere and bulk soil.

**Figure 6:**
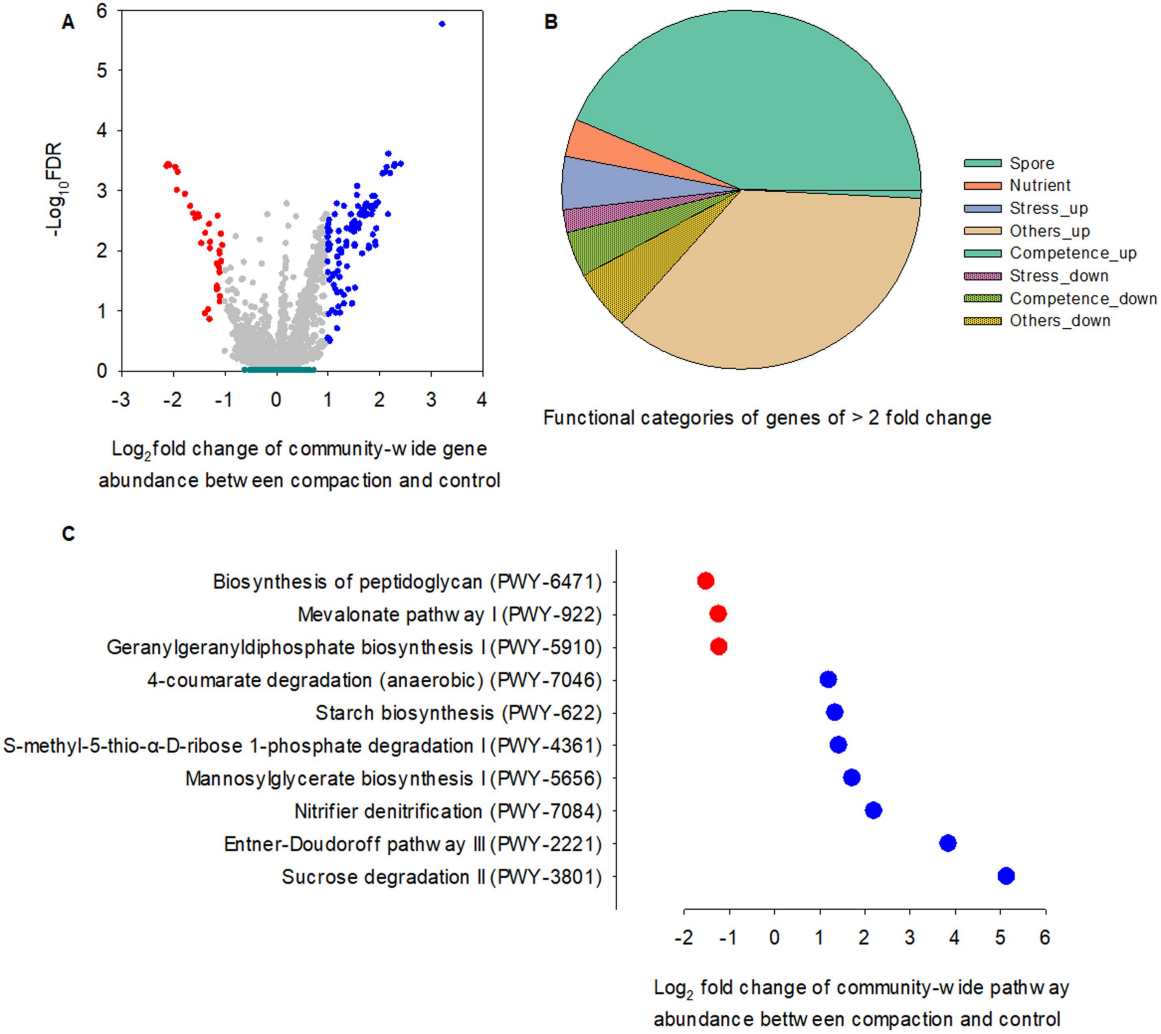
Comparisons of PICRUSt-predicted KO genes and pathways between two foot traffic treatments. **(A)** The volcano plot depicts the significance and magnitude of changes in the relative abundance of KO genes between simulated foot traffic at three time per week from September to November, mimicking weekly six professional football games and no foot traffic treatment. Dark green represents 24 replicates for the rhizosphere and 24 replicates for bulk soil (i.e., 2 grass species × 12 cultivars). Gray, red and blue represent 24 replicates (i.e., 2 grass species × 12 cultivars) from the root endosphere for no difference, under-representation and over-representation, respectively, when comparing simulated foot traffic in nominator with no foot traffic in denominator. **(B)** The pie chart summarizes under- and over-represented KO genes in the root endosphere, accounting for ~3.4% of total number of functional genes, into different functional categories. **(C)** The scatter plot represents pathways differing in the relative abundance between simulated foot traffic and no foot traffic in the root endosphere. Each dot represents the mean value of 24 data points (i.e., 2 grass species × 12 cultivars).

Foot traffic showed little impact on the relative abundance of microbial functional genes in the rhizosphere and bulk soil (Figure 6A), However, 3.4% of total microbial functional genes in the root endosphere were significantly affected, of which ~88% were over-represented under foot traffic, with the majority of changes belonging to genes contributing to sporulation/spore germination and nutrient transports (Figures 6A,B). Most stress-responsive genes were promoted by foot traffic, except for *spxA*, a stress response regulator, and *cshB*, a cold shock gene. Genes involved in competence were in sharp contrast, being over-represented under no foot traffic.

A total of ten biological pathways were found to vary with foot traffic treatments, of which 70% was over-represented under foot traffic, including sucrose and glucose degradation (PWY-3801, PWY-2221) and nitrifier denitrification (PWY-7084) (Figure 6C). Mannosylglycerate biosynthesis I, the pathway (PWY-5656) for producing 2-O-(*α*-D-mannosyl)-D-glycerate, a compatible solute to protect against osmotic pressure, was also promoted by foot traffic. Other pathways that were enhanced under foot traffic included S-methyl-5-thio-α-D-ribose 1-phosphate degradation (PWY-4361), starch biosynthesis (PWY-622), and 4-coumarate degradation (PWY-7046). PWY-4361, known as the universal methionine salvage pathway, is crucial to regain methionine, an essential amino acid and the precursor of a plant hormone and pheromone, ethylene. Starch and glycogen are major reservoirs of carbon and energy for organisms and help organismal survival under stress. Those polymers can accumulate in cells when carbon in the environment is in excess or when organismal growth is limited by nutrients, such as nitrogen. Therefore, significant differences in the relative abundance of PWY-622 between foot traffic treatments signified foot traffic-associated changes in root physiology, biochemistry, and metabolites. This was further supported by differences in PWY-7046, given that aromatic compounds, e.g., coumarate from plants, are carbon sources for microbes, and the level of coumarate can be associated with stress, such as water limitation. By contrast, foot traffic suppressed the relative abundance of pathways involved in cell wall biosynthesis (PWY-6471, PWY-922, PWY-5910), suggesting microbial vulnerability to exogenous stress.

## 4 Discussion

To our knowledge, this was the first study showing that foot traffic could exert profound impacts on the composition and function of the microbial community within plant roots. This study also demonstrated that along the bulk soil-rhizosphere-root endosphere continuum, the effect of foot traffic was most pronounced in the root endosphere. Further, this study shed insight into how root endophytes could help plants cope with foot traffic-induced stress. While foot traffic could lead to soil compaction, plant tearing, and wear, their effects on structuring the microbiome in turfgrass roots should not be overlooked.

### 4.1 Foot traffic affected the composition but not the diversity of microbiomes

Soil is a porous environment with resources (e.g., carbon, nutrients, water, and air) being distributed heterogeneously. This creates various micro-niches to support the co-existence of morphologically, physiologically, and phylogenetically diverse microbes. Therefore, it is not surprising to observe that both bacterial and fungal diversity were much greater in the bulk soil than in the rhizosphere and root endosphere (Figure 1), where phytochemicals might have a strong selection pressure to favor the proliferation of a subset of the microbiome, e.g., copiotrophs (Bulgarelli et al., 2012; Lundberg et al., 2012; Ling et al., 2022). Indeed, corroborated with a meta-analysis showing that Proteobacteria were enriched in the root zone than the bulk soil, we also found that this bacterial phylum became more abundant from the bulk soil and rhizosphere to root endosphere (Figure 3A).

Foot traffic equivalent to the intensity of six professional football games per week from September to November had little influence on the microbial diversity of the bulk soil, which aligned with the results of Hartmann et al. (2014), showing relatively stable microbial diversity under machinery traffic-induced slight soil compaction, although severe soil compaction increased both bacterial and fungal diversity. We also found that microbial diversity in the rhizosphere and root endosphere was kept unaffected by foot traffic. These findings suggested that foot traffic-induced perturbation was not strong enough to considerably shape the degree of heterogeneity in resources and space, despite quite different ecological factors dominating the assembly of the microbiome among the three microhabitats. It should be mentioned that similar microbial diversity indicators did not necessarily mean no variations in the composition of the microbial community with foot traffic.

We did observe the effects of foot traffic on bacterial and fungal communities, besides microhabitats-dominant community separation (Figure 2). However, the significance of foot traffic relative to edaphic factors in structuring the microbial community was unequal among microhabitats and varied with microbial domains, i.e., bacteria versus fungi, indicating different mechanistic modes of action. In the bulk soil, foot traffic was expected to cause compaction and reduce the volume of macropores. As a consequence, fungi were more likely affected than bacteria due to their filamentous structure and preference for well-aerated pores (Otten et al., 1999; Harris et al., 2003). This appears to explain our observation that foot traffic was more important in modulating the fungal community than the bacterial community in the bulk soil. By contrast, the root endosphere is a liable organic-rich environment, and changes in quantity and quality of phytochemicals likely affect bacteria more strongly than often-considered oligotrophic fungi. Foot traffic could lead to considerable grass wear and tearing, severely damaging photosynthesis and, in turn, the allocation of photosynthates to the root system as well as the rate of root tissue turnover (Nguyen, 2003; Haichar et al., 2014). This, together with compaction-induced constraints on nutrient and water uptake, perhaps served as a primary driver to regulate the community composition of copiotrophic bacteria under foot traffic.

Different drivers for the assembly of bacterial versus fungal communities within and outside of roots were further exemplified by pH impacts. While the study site was acidic, the pH of individual soil samples varied up to ~1.5 units (Table 1). Because fungi were more tolerant to acidic environments than bacteria, pH was not a significant driver to the soil fungal community, but rather the soil bacterial community. While endophytic microbes may live in apoplast, cell wall-plasma membrane peri-space, or even cytosol, internal pH remains relatively stable at these subcellular-specific values, meaning roots were likely pH-homeostasis (Martinière et al., 2018; Frene et al., 2024). Hence, environmental pH showed little influence on the community composition within root tissues but mainly in bulk soil and the rhizosphere. Similarly, moderate changes in soil moisture affected little on the bacterial and fungal communities within roots, but significantly outside of roots. This suggested that grass roots served as a buffer to reduce, to some degree, the oscillation of environmental factors. However, this was not the case for soil inorganic N that varied in the range of ~9–35 mg N kg^−1^ soil among individual samples (Table 1). Soil inorganic N significantly influenced the composition of the bacterial community, irrespective of microhabitats (Figure 2), implying that root N uptake and, thus, internal N content varied considerably with the availability of soil inorganic N. No significant effects on the fungal community was likely due to lower N demand than the bacterial community since fungi has a much wider C:N ratio than bacteria.

While soil pH, moisture, and inorganic N all differed significantly between foot traffic treatments (Table 1), foot traffic effects on the microbial community were perhaps not through one but the multiplication of these factors. This is because neither the magnitude nor the direction of foot traffic effects was paralleled with any of the soil properties, as visualized in db-RDA (Figure 2). The strongest endosphere effects of foot traffic indicated that its modulation on the microbial community was largely through grass responses and associated changes in phytochemicals.

### 4.2 Sensitive taxa to foot traffic across the bulk soil-rhizosphere-root endosphere continuum

Large divergence in microbiome composition and structure among the three microhabitats resulted most likely from the selection, by microhabitat-unique environments, of microbes having specific traits. Within root tissues, microbial ability to move, degrade plant cell-wall compounds and scavenge reactive oxygen species has been suggested to help better colonization of microbes (Liu et al., 2017). The topmost abundant bacterial phyla are often Proteobacteria, followed by Actinobacteria and Bacteroidetes, whereas Acidobacteria and Gemmatimonadetes are under-represented (Liu et al., 2017). Such a pattern of bacterial distributions also appeared in this and previous studies of the turfgrass root microbiome across various ecological settings (Xia et al., 2021; Hu et al., 2023a,b), further highlighting the presence of universal drivers and principles for the assembly of the root microbiome. Still, Chloroflexi were noticed to be sensitive to foot traffic, irrespective of microhabitats (Figure 3).

Similar to Actinobacteria, Chloroflexi are monoderm, filamentous, and spore-forming bacteria and are thought to tolerate various stressors, including drought and starvation. While not much is known about their ecological niche, a few studies showed that Chloroflexi and Actinobacteria co-responded to environmental stress (Santos-Medellin et al., 2017; Edwards et al., 2018). Drought, for example, enriched both Actinobacteria and Chloroflexi. In our previous studies, however, severe drought by no irrigation promoted Actinobacteria in grass roots but reduced Chloroflexi (Hu et al., 2023a,b), suggesting that Actinobacteria and Chloroflexi differed to some degree in terms of resistance to a stressor of different severities. This appeared to be supported by a recent investigation of the soil microbiome in different geographic areas in Australia (Maisnam et al., 2023). The authors showed that Chloroflexi and Actinobacteria were dominant at semi-arid and arid sites, respectively. Nonetheless, despite not being manifested at the phylum level, a genus detected to be significant between foot traffic treatments belonged to the phylum Actinobacteria in the bulk soil and the rhizosphere. What was the driver(s) behind foot traffic to benefit Chloroflexi or the member of Actinobacteria?

Soil compaction has been documented to affect both bacterial and fungal community composition as a result of compaction-induced low air permeability (i.e., low oxygen availability) (Hartmann et al., 2014; Longepierre et al., 2021). They found that some members of Proteobacteria and Firmicutes were better at adapting to low oxygen than other microbes and, therefore, positively responded to soil compaction. On the contrary, soil compaction reduced the relative abundance of Actinobacteria. Obviously, their data were not in line with ours (Figures 3, 4), suggesting that factors other than compaction-induced air permeability were more important in structuring the soil microbial community. Our observations likely stemmed from foot traffic-induced root turnover and associated changes in soil organic carbon biochemistry, promoting some decomposers over others. Further foot traffic-induced changes in habitat pore space might modulate the interaction among selected decomposers. This was because foot traffic responsive Chloroflexi and members of Actinobacteria have been shown to co-respond to soil particle/pore size distribution and contribute to the decomposition of recalcitrant organic matter (Seaton et al., 2020; Xia et al., 2020, 2022).

The inconsistency between our findings and those of others (Hartmann et al., 2014; Longepierre et al., 2021) is unlikely due to an excessively long sampling interval after foot traffic, during which the microbiome may have partially recovered. Machinery traffic-induced soil compaction has been found not to affect the soil microbiome immediately but a few months later; more noticeably, microbial taxa sensitive to soil compaction showed a very low capability of resilience (Longepierre et al., 2021). Actually, foot traffic-induced soil compaction from last fall persisted, as indicated by still significantly lower soil water content 1 year later (Table 1). This, together with compaction-induced possible low air permeability would reduce microbial activity for decomposition of soil organic matter, releasing less inorganic N to soil (Table 1). Our findings implied that foot traffic exerted lasting effects on soil physical properties and, accordingly, microbial activity. However, the compositional changes of the microbiome did not mirror foot traffic-induced changes in soil physical properties, which was dissimilar to the results of other studies (Hartmann et al., 2014; Longepierre et al., 2021). Perhaps, this was due to differences in plant cover. In other studies, machinery traffic put pressure directly on soil but not on plants, whereas foot traffic on turf caused grass tearing and wear. In addition, perennial turf has dense roots and high organic matter as well, especially in the top 10 cm depth, which could help minimize foot-traffic induced soil compaction.

### 4.3 Modulations in the potential function of the root microbiome by foot traffic

The gene profile of the root endosphere was quite different from that of bulk and rhizosphere soil, highlighting that microbiomes of different metabolic strategies were selected to cope with respective environmental cues in the bulk soil, rhizosphere, and root endosphere. The relative ‘harsh’ conditions in the soil, such as more frequent and large fluctuations in moisture, temperature, and available nutrients, favor microbes capable of shifting metabolism quickly from the high to low, and even dormant state. As such, compared to the root endosphere, bulk and rhizosphere soil possessed more abundant sporulation- and spore germination genes. Soil microbes use organic matter as carbon and energy sources and are heavily involved in the transformations of nutrients of different forms or species, specifically nitrogen. This was mirrored well in the greater relative abundance of nitrogen mineralization and nitrification in soil than in the root endosphere. On the contrary, roots are a carbon- and nutrient-rich environment for microbes. Increasing evidence supports that plants and associated microbes function as a holobiont for sustaining growth as well as coping with the environment. Despite intensive management to create conditions that help turfgrass survival and growth under often-naturally suboptimal conditions, still, roots harbored diverse and putatively beneficial microbes.

It was beyond our objective to extensively compare microbial community functions among the three microhabitats. However, two groups of genes or gene clusters that were overrepresented in the root endosphere need to be stressed. One group was related to toxin production and immunity buildup that have been suggested to suppress soil-borne pathogens via antibiotics and to enhance plant growth via systemic resistance and metal-chelation that has an influence on nutrient availability (Edgar and Bibi, 1997; Stock et al., 2000; Ramette et al., 2003; Ryu et al., 2003 Rudrappa et al., 2010; Rijavec and Lapanje, 2016). Another group included genes encoding stress signals and hormone production to regulate metabolic, physiological, and developmental processes in plants (Rijavec and Lapanje, 2016). Particularly, *AcdS* to encode ACC deaminase has been drawn attention due to its degradation of the precursor of phytohormone ethylene. Inoculating microbes of *AcdS* capability into plants has been shown to reduce ethylene overproduction and accordingly improve plant growth (Reed et al., 1995; Sergeeva et al., 2006; Farwell et al., 2007; Zhang et al., 2008; Jung et al., 2018). It is worth noting that the gene profile was predicted based on microbial taxonomic composition. The reliability of this inference-based tool for environmental samples has been a concern, specifically pertaining to functional genes in the metabolism of terpenoids, polyketides, xenobiotics biodegradation as well as signaling molecules (Sun et al., 2020; Djemiel et al., 2022). Still, this tool has been demonstrated to be robust in inferring shifts in metabolic strategies at the community scale (Raes et al., 2021). Here, we showed that the divergence of gene profiles between soil and the root endosphere was in line with the general notion that the microbiota in soil and roots are responsible for nutrient transformations and plant health, respectively, implying that this marker gene-based prediction tool was authentic in the turf environment.

Foot traffic could affect plant growth through multiple modes of action. One was through soil compaction. The underlying mechanism would likely be that soil compaction lowered gas diffusion and therefore, the phytohormone (e.g., ethylene) accumulated around root tips, causing roots to decrease in elongation to cope with compacted soil (Frene et al., 2024). Further, this would reduce water uptake and result in plant dehydration, triggering molecular-level responses to enhance root adaptation under stress (Kim et al., 2024). In concert, the root-associated microbiota as a whole responded to changes and helped modulate phytohormones and signaling molecules. As a result, we expected to observe variations related to root growth regulation and osmotic pressure. We did find that the nitrifier denitrification pathway (PWY-7084), which controls the production and consumption of nitric oxide (NO) became abundant by foot traffic. The NO is a key signaling molecule that enhances root development and adaptation under stress by promoting lateral root formation and defense against oxidative stress. We also observed that the relative abundance of mannosylglycerate biosynthesis (PWY-5656) was promoted by foot traffic. Mannosylglycerate, a compatible solute, is known for its superior protein-stabilizing properties, protecting enzymes against inactivation caused by osmotic stress (Ramos et al., 1997; Shima et al., 1998; Lamosa et al., 2000; Borges et al., 2002; Longo et al., 2009, Santos et al., 2011). Apparently, our data suggested the sensitivity of root endophytes to intensive foot traffic and possibly-induced soil compaction and their contributions to healthy plants under stress.

Another way by which foot traffic affected plant health was through plant tearing and wear. The decline in photosynthesis, together with soil compaction-induced root dieback, would accelerate the turnover of root tissues. This would increase the release of plant-derived carbohydrates, such as sucrose and glucose. This mode of action by foot traffic was suggested by the over-representation of sucrose and glucose degradation pathways in bacteria. This was also implied by the greater relative abundance of the degradation pathway (PWY-7046) of 4-coumarate, a constituent of grass cell walls via eater-linked to lignin. Further, the over-representation of starch or glycogen synthesis pathway (PWY-622) suggests that root tissue turnover flushed root endophytes with extra organics and promoted microbial carbon storage.

## 5 Conclusion

Foot traffic on sports turf might not considerably reduce soil macro-pores and total porosity as expected, because extensive root tissues and relatively high soil organic matter content could, to some degree, alleviate soil compaction. Yet, a moderate decline in soil void space seemed to occur, lowering soil water content and possibly air permeability. Soil microbial activity appeared to be more sensitive to the modest alteration in soil physical properties than was the diversity and composition of the soil microbiome. The root bacteriome responded to foot traffic in composition as well as in the genetic potential (genes and pathways). The significant yet a few changes in the gene profile and pathways indicated that plants might rely on the activation and persistence of specific microbial functions rather than broad changes in microbial community composition to cope with and recover from prolonged stress. Further research using whole genome sequencing, e.g., shotgun metagenomic sequencing, is needed to more reliably estimate microbial functional changes under stress. This will better advance our knowledge highlighting how the root microbiome helps turfgrass to cope with foot traffic.

## Data Availability

The datasets presented in this study can be found in online repositories. The names of the repository/repositories and accession number(s) can be found in the article/Supplementary material.
