# Supplementary material for: Foot traffic on turf primarily shaped the endophytic bacteriome of the soil-rhizosphere-root continuum
Source: Front Microbiol. 2025 Apr 9;16:1488371. doi: 10.3389/fmicb.2025.1488371 (PMC12014648; doi:10.3389/fmicb.2025.1488371)
Supplement: Supplementary file 1 [file Table_1.docx]

Supplementary data

**Foot traffic on turf primarily shaped the endophytic bacteriome of the soil-rhizosphere-root continuum**

Sayada Momotaz Akther, Jialin Hu^#^, Grady Miller, and Wei Shi^*^

Department of Crop and Soil Sciences, North Carolina State University, Raleigh, NC 27695, USA

^*^Corresponding author: Wei Shi, wei_shi@ncsu.edu

**^#^**Current address: AgriLife Research, Texas A&M University, College Station, TX 77843, USA.

Table S1. PERMANOVA test results of examining if bacterial and fungal beta-diversities were significantly affected by traffic treatments (traffic and non-traffic), microhabitats (bulk soil, rhizosphere, and root endosphere), and their interactions. A generalized linear mixed effects model with random effect of cultivars/genotypes nested within species was used for the statistical analysis of microbial diversity and composition.

|  | Df | SumsOfSqs | F.Model | R^2^ | Pr(>F) |
| --- | --- | --- | --- | --- | --- |
| Bacteria |  |  |  |  |  |
| Treatment (T) | 1 | 0.359 | 1.398 | 0.008 | 0.105 |
| Habitats (H) | 2 | 12.548 | 24.449 | 0.264 | 0.001 *** |
| T x H | 2 | 0.479 | 0.933 | 0.010 | 0.453 |
| Residuals | 133 | 34.129 |  | 0.718 |  |
| Total | 138 | 47.515 |  | 1.000 |  |
| Fungi |  |  |  |  |  |
| Treatment (T) | 1 | 0.450 | 1.505 | 0.010 | 0.069 |
| Habitats (H) | 2 | 7.406 | 12.374 | 0.151 | 0.001 *** |
| T x H | 2 | 0.355 | 0.593 | 0.007 | 0.995 |
| Residuals | 137 | 41.001 |  | 0.833 |  |
| Total | 142 | 49.213 |  | 1.000 |  |

^#^Df, degree of freedom; SumsOfSqs, sum of squares; Pr(>F), *P*-value for F statistics
